# BLTR1 in Monocytes Emerges as a Therapeutic Target For Vascular Inflammation With a Subsequent Intimal Hyperplasia in a Murine Wire-Injured Femoral Artery

**DOI:** 10.3389/fimmu.2018.01938

**Published:** 2018-08-28

**Authors:** Seung E. Baek, So Y. Park, Sun S. Bae, Koanhoi Kim, Won S. Lee, Chi D. Kim

**Affiliations:** ^1^Department of Pharmacology, School of Medicine, Pusan National University, Yangsan, South Korea; ^2^Gene and Cell Therapy Research Center for Vessel-Associated Diseases, Pusan National University, Yangsan, South Korea

**Keywords:** high mobility group box 1, 5-lipoxygenase, leukotriene B4 receptor, monocyte-to-macrophage differentiation, vascular restenosis, intimal hyperplasia

## Abstract

Given the importance of high-mobility group box 1 (HMGB1) and 5-lipoxygenase (5-LO) signaling in vascular inflammation, we investigated the role of leukotriene signaling in monocytes on monocyte-to-macrophage differentiation (MMD) induced by HMGB1, and on vascular inflammation and subsequent intimal hyperplasia in a mouse model of wire-injured femoral artery. In cultured primary bone marrow-derived cells (BMDCs) stimulated with HMGB1, the number of cells with macrophage-like morphology was markedly increased in association with an increased expression of CD11b/Mac-1, which were attenuated in cells pre-treated with Zileuton, a 5-LO inhibitor as well as in 5-LO-deficient BMDCs. Of various leukotriene receptor inhibitors examined, which included leukotriene B4 receptors (BLTRs) and cysteinyl leukotriene receptors (cysLTRs), the BLTR1 inhibitor (U75302) exclusively suppressed MMD induction by HMGB1. The importance of BLTR1 in HMGB1-induced MMD was also observed in BMDCs isolated from BLTR1-deficient mice and BMDCs transfected with BLTR1 siRNA. Although leukotriene B4 (LTB4) had minimal direct effects on MMD in control and 5-LO-deficient BMDCs, MMD attenuation by HMGB1 in 5-LO-deficient BMDCs was significantly reversed by exogenous LTB4, but not in BLTR1-deficient BMDCs, suggesting that LTB4/BLTR1-mediated priming of monocytes is a prerequisite of HMGB1-induced MMD. *In vivo*, both macrophage infiltration and intimal hyperplasia in our wire-injured femoral artery were markedly attenuated in BLTR1-deficient mice as compared with wild-type controls, but these effects were reversed in BLTR1-deficient mice transplanted with monocytes from control mice. These results suggest that BLTR1 in monocytes is a pivotal player in MMD with subsequent macrophage infiltration into neointima, leading to vascular remodeling after vascular injury.

## Introduction

Vascular endoluminal interventional procedures injure vascular walls, and result in the release endogenous damage-associated molecular patterns (DAMPs) (1, 2). Of the various DAMP proteins, high mobility group box 1 protein (HMGB1) has emerged as an important regulator of inflammatory responses resulting from tissue injury (3–5), and been implicated as an active player in vascular inflammation with resultant intimal hyperplasia after arterial injury (6). In a previous study, we found HMGB1 enhanced monocyte-to-macrophage differentiation (MMD) and resultant vascular inflammation in injured vasculature (7), and thus, we suggested coordinated relationships exist between local vascular injury and pattern recognition receptor-related signals in the process of vascular inflammation.

Monocyte recruitment to injured tissues, their subsequent transformation into macrophages, and the overproduction of inflammatory cytokines are major steps in the process of vascular inflammation (8, 9). These sequential events stimulate vascular smooth muscle cell (VSMC) proliferation and extracellular matrix deposition in neointima, which result in intimal hyperplasia and vascular occlusion (10). Furthermore, several key proteins involved in the leukotriene cascades, such as, 5-lipoxygenase (5-LO) and arachidonate 5-lipoxygenase activating protein (FLAP), and leukotriene (LT) receptors are highly expressed in human atherosclerotic plaque (11–13), which suggests their potential involvements in vascular inflammation.

Previous studies have reported genetic targeting of 5-LO reduced lesion size in atherosclerosis prone mouse strains (14–16). Likewise, in a previous study, we found 5-LO importantly contributed to the development of atherosclerosis by increasing the expressions of adhesion molecules on monocytes, and thus, increasing monocyte adhesion to vascular endothelium (17). In FLAP-deficient mice, neointima hyperplasia in injured arteries was significantly attenuated by reducing inflammatory cytokine release from FLAP-deficient macrophages (18), which suggested 5-LO in macrophages plays a pivotal role in vascular inflammation. However, although 5-LO in inflammatory cells has been proposed to be an important player in the development of vascular inflammation (12, 19), the importance of 5-LO signaling pathways in monocytes in vascular inflammation with subsequent vascular remodeling in injured vasculatures remains unclear.

Leukotrienes (LTs) are considered to mediate inflammatory responses in various cardiovascular diseases characterized by vascular inflammation (20). LTs exert their actions via four subclasses of receptors, such as, BLT1 and BLT2 (receptors for LTB4), and CysLT1 and CysLT2 (receptors for cysteinyl-leukotrienes) (21). Previous studies have implicated LT receptor activation in atherogenesis and vascular remodeling after angioplasty (21, 22), and studies on the genetic and pharmacological targeting of BLTR1 in atherosclerotic mouse strains further supported the involvement of leukotriene-signaling in vascular inflammation (23–25). However, the precise role of BLTR1 signaling in monocytes in the process of vascular inflammation remains unclear.

In a previous study, we described the importance of 5-LO in monocytes during vascular inflammation (7). However, our incomplete understanding of how 5-LO signaling pathways in monocytes contribute to vascular inflammation explains the incapability of current treatments to prevent vascular remodeling in the injured vasculatures. Given the importance of HMGB1 and 5-LO signaling in monocytes during vascular inflammation, we investigated the role of leukotriene signaling in monocytes on MMD induced by HMGB1. To further determine the contribution of 5-LO signaling in monocytes in macrophage infiltration into neointima lesions, we also investigated the importance of BLTR1 signaling in monocytes in vascular inflammation and subsequent intimal hyperplasia using BLTR1-deficient mice and BLTR1-deficient mice transplanted with monocytes from WT mice.

## Materials and methods

### Ethics statement and animals

All experiments involving animals conformed with the Guide for the Care and Use of Laboratory Animals published by the US National Institute of Health (NIH Publication No. 85-23, 2011 revision), and all animal-related experimental protocols were approved by the Pusan National University Institutional Animal Care and Use Committee of the College of Medicine (PNU-2016-1310). Genotyping, including that of 5-LO-deficient mice and BLTR1-deficient mice, was performed by PCR using a protocol provided by the Jackson Laboratory (Harlan Nossan, ITA). Wild-type (WT) control mice (C57BL/6J) were purchased from the Jackson Laboratory. Animals were housed in an air-conditioned room at 22–25°C and kept under a 12-h light/dark cycle. Food and water were provided *ad libitum*.

### Vascular injury models and blood flow measurement

C57BL/6J (WT), 5-LO-deficient and BLTR1-deficient male mice (7 wk-old) were subjected to right femoral artery injury using a 0.25 mm diameter angioplasty guidewire under chloral hydrate (450 mg/kg, intraperitoneal injection) anesthesia and aseptic conditions, as previously described (26). The adequacy of anesthesia was confirmed by response to toe pinch. Wire-injured femoral arteries were harvested from mice euthanized by carbon dioxide insufflations and cervical dislocation, and then cross sectioned (4 μm). Tissue sections were stained with hematoxylin and eosin (H&E) and immunohistological marker antibodies. Femoral arterial blood flow was measured using a laser Doppler perfusion imaging (LDPI) analyzer (Moor Instruments, Devon, UK) at 0, 1, 2, 3, and 4 wks after femoral artery injury. The changes in blood flow were calculated using the colors of histogram pixels.

### Chemicals and antibodies

Zileuton and alpha-smooth muscle actin (α-SMA) antibody were purchased from Sigma-Aldrich (St. Louis, MO, USA). LTB4, U75302 and REV5901 were from Cayman Chemical Inc (Ann Arbor, MI, USA). MK 886 was purchased from EMD Serono (Rockland, MA, USA), HMGB1 from R&D systems (Minneapolis, MN, USA), CD11b antibody from Abcam (Cambridge, MA, USA), and BLTR1 antibody from Biorbyt (Cambridge, UK). CD36, CD14, β-actin, and 5-LO antibodies were purchased from Santa Cruz Biotechnology (Santa Cruz, CA, USA), R-phycoerythrin PE-conjugated mouse anti-human CD11b/Mac-1 antibody and PE-conjugated mouse IgG isotype control antibody from BD (San Diego, CA, USA). Horseradish peroxidase (HRP)-conjugated IgG antibody was used as the secondary antibody from Santa Cruz Biotechnology. Restriction enzymes were purchased from Promega (Madison, WI, USA). 5-LO and BLTR1 siRNA oligonucleotides were synthesized by Bioneer (Daejeon, ROK). siRNA molecules were transfected into cells using Lipofectamine 2000 siRNA transfection reagent (Invitrogen, Carlsbad, CA, USA). PCR primers were from Bioneer.

### Isolation of bone marrow-derived cells and culture

Bone marrow derived cells (BMDCs) were isolated from mice (7 wks, male) euthanized by carbon dioxide insufflation and cervical dislocation. Briefly, after bone marrow cells were harvested from femurs and tibiae, red blood cells were lysed using lysing buffer (Sigma-Aldrich) and incubated in RPMI1640 containing 10% heat-inactivated fetal bovine serum (FBS) for 24 h. Non-adherent cells were harvested and centrifuged at 1300 rpm for 10 min, and the cell pellets so obtained were washed twice with PBS and resuspended in RPMI 1640 containing 10% FBS. BMDCs were maintained in RPMI 1640 containing 10% FBS and antibiotic-antimycotic (Life technologies, Carlsbad, CA, USA) at 37°C. Cells (5 × 10^5^/mL) were seeded and cultured for 24 h in complete medium for further experiments.

### Flow cytometric analysis

BMDCs were resuspended in fluorescence activated cell sorter (FACS) buffer (PBS containing 1% FCS and 0.05% NaN_3_), to assess the surface expression of CD11b/Mac-1 protein. Cells were incubated with a FcR blocker to block non-specific antibody binding, and then incubated with PE-conjugated anti-mouse CD11b antibody (1:500). Analysis was performed using a FACS Calibur and CELLQUESTPRO software BD, and 1 × 10^4^ cells were recorded per sample. Live cells were gated based on size (FSC) and granularity (SSC), and then CD11b/Mac-1 expression was analyzed. Fluorescence was analyzed by FACS as described above.

### Reverse transcription-PCR analysis

Total RNA was isolated from cells using QIAzol (Qiagen, Hilden, Germany) and reverse transcribed into cDNA using the Improm-II Reverse Transcription System (Promega). cDNA amplification was performed using primers specific for 5-LO (forward, 5′-ATTGCCATCCAGCTCAACCAAACC-3′; reverse, 5′-TGGCGATACCAAACACCTCAGACA-3′). 5-LO mRNA levels in BMDCs were quantified by RT-PCR using GAPDH mRNA as the internal standard. Relative intensities were expressed as fold changes vs. GAPDH.

### Western blot analysis

BMDC lysates were prepared in ice-cold lysis buffer, and equal amounts of proteins were separated on 8~10% polyacrylamide gel under reducing conditions, and then transferred to nitrocellulose membranes (Amersham-Pharmacia Biotech, Piscataway, NJ, USA). Membranes were blocked with 5% skim milk in TBST and incubated overnight with primary antibody (1:1000) in 5% skim milk. Blots were washed with TBST, incubated with HRP-conjugated secondary antibody for 2 h, and developed using ECL Western blot detection reagents (Amersham-Pharmacia Biotech). Membranes were re-blotted with anti-β-actin antibody (Santa Cruz Biotechnology) as an internal control. Signals from bands were quantified using US-SCAN-IT gel 5.1 software (Silk Scientific, Orem, Utah, USA). Results were expressed as relative densities.

### Quantitative real-time reverse transcription analysis

Total RNA was isolated from cells using QIAzol (Qiagen) and reverse transcribed into cDNA using the Improm-II Reverse Transcription System (Promega). BLTR1 gene expression was determined by real-time PCR using 1 ng of reverse-transcribed cDNA and a LightCycler 96 system equipped with LightCycler DNA Master SYBR Green I (Roche Molecular Biochemicals, Mannheim, Germany). PCR was performed under the following conditions: 95°C for 10 min followed by 50 amplification cycles of 95°C for 10 s, 45°C for 10 s, and 72°C for 10 s. Amplification efficiencies were calculated and normalized with respect to mouse GAPDH. The PCR primers used were as follows: forward, 5'-TTACCACCTGGTGAACCTGGTGGAA-3'; reverse, 5'-TTCGAAGACTCAGGAATGGTGGAG-3'. Quantities were calculated using standard curves.

### Measurement of LTB4 production

LTB4 production was measured in extracellular medium using an LTB4 assay kit (Cayman Chemical Inc., Ann Arbor, MI, USA) according to the manufacturer's instructions. Briefly, BMDCs were stimulated with HMGB1 (100 ng/ml), and LTB4 levels in concentrated media were quantified by ELISA (Bio-Tek Instrument Inc., Winooski, VT, USA).

### Preparation of BLTR1 siRNA and *in vitro* transfection

Small interfering RNA (siRNA) for BLTR1 and scrambled siRNA (negative control) were designed and synthesized using a Silencer^TM^ siRNA construction kit purchased from Bioneer. The sequences of BLTR1 siRNA and scrambled siRNA were 5′-GAUCUGCGCUCCGAACUAUdTdT-3′ and 5′-AUAGUUCGGAGCGCAGAUCdTdT-3′, respectively. For siRNA transfection, cells were seeded and transfected with BLTR1 siRNA using Lipofectamine 2000 (Invitrogen, NY, USA) according to the manufacturer's protocol. Transfection efficiencies were monitored using a fluorescent oligonucleotide (BLOCK-iT Fluorescent Oligo; Invitrogen) and estimated to be between 80 and 90%.

### Immunofluorescence analysis

Wire-injured femoral arteries were harvested and serial paraffin sections (4 μm) of femoral arteries were incubated with mouse-anti α-SMA (1:400) and rabbit-anti CD36 (1:200) antibodies. Alexa488-conjugated IgG and Alexa594-conjugated IgG (Abcam) were used to detect immunofluorescence signals for α-SMA and CD36, respectively. After nuclei were visualized by staining with 0.1 μg/ml diamidino-2-phenylindole (DAPI), slides were mounted in Vectashield. Fluorescence images were visualized by scanning confocal microscopy (LSM 510, Carl Zeiss, Oberkochen, Germany), and analyzed by National Institutes of Health (NIH) image software (Image J, NIH, USA).

### Transplantation of bone marrow-derived monocytes

BMDCs were harvested from the femurs and tibiae of mice (7 wks, male), which had been euthanized by carbon dioxide insufflation and cervical dislocation, and bone marrow-derived monocytes (BMDMs, CD11b-positive cells) were then separated using MACS technology (Miltenyi, Bergisch Gladbach, GER) using a standard procedure. BMDCs were then stained with fluorochrome-labeled monoclonal anti-CD11b, sorted using a BD ARIAIII cell sorter (Becton Dickinson, San Jose, CA, USA), washed, and resuspended at 1 × 10^7^ cells/ml. Recipient BLTR1-deficient mice were administered 1 × 10^7^ BMDMs per mouse by tail vein injection. The expressions of BLTR1 mRNA and protein in peripheral blood monocytes (PBMCs) isolated from three groups of BMDMs-transplanted mice (WTWT mice, WT monocytes into WT mice; KOKO mice, BLTR1-deficient monocytes into BLTR1-deficient mice; and WTKO mice, WT monocytes into BLTR1-deficient mice) were determined by Real Time PCR and immunocytochemistry, respectively.

### Statistical analysis

Results were expressed as means ± SEMs. One-way analysis of variance (ANOVA) followed by Turkey's multiple comparison test was used to determine the significance of differences. Statistical significance was accepted for *P* values < 0.05.

## Results

### A role for 5-LO in MMD induced by HMGB1

**Figure 1:**
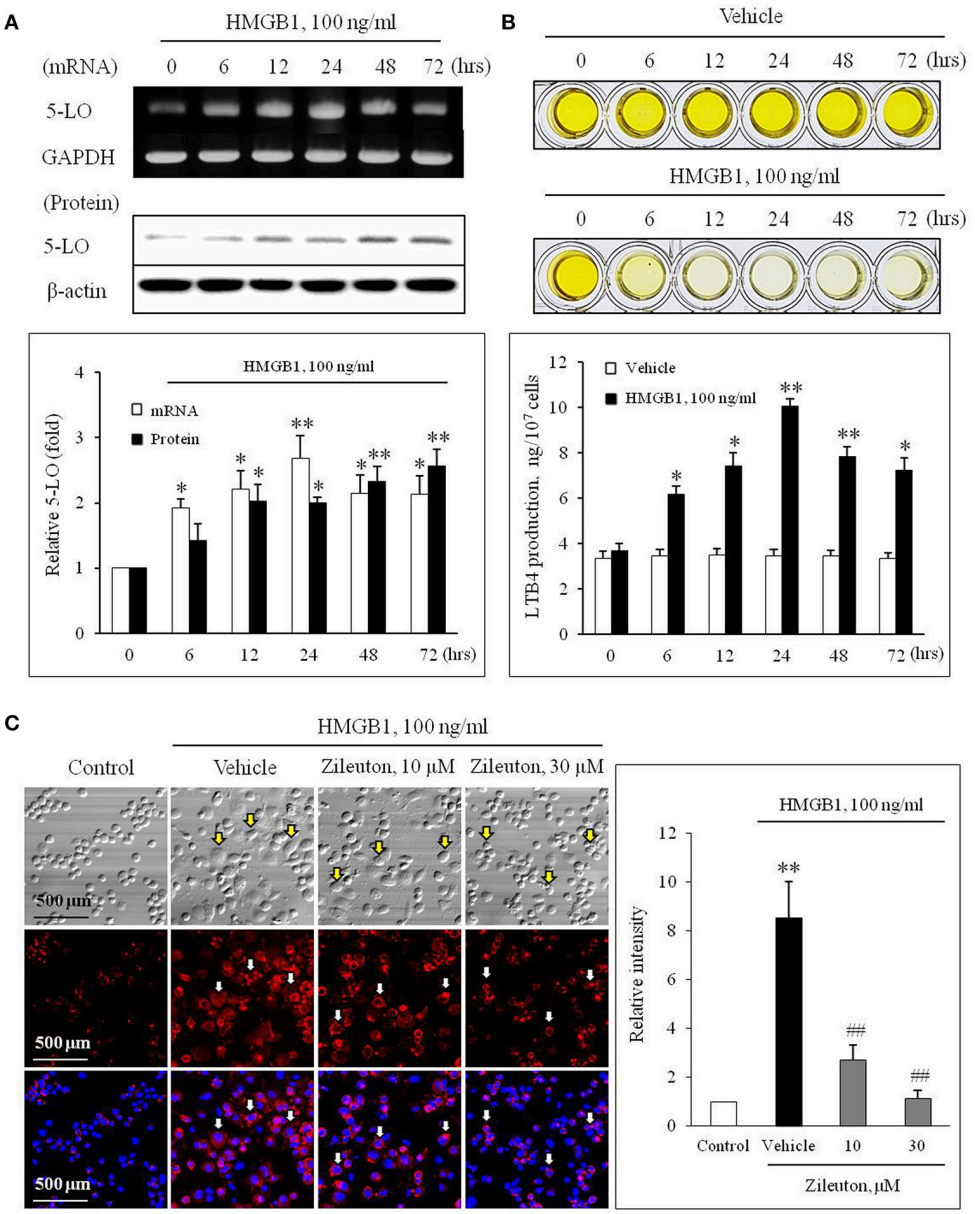
Role of 5-LO in monocytes on monocyte-to-macrophage differentiation (MMD) induced by HMGB1. **(A)** Time-courses of the expressions of 5-LO mRNA and protein in BMDCs stimulated with HMGB1 (100 ng/ml) were determined using RT-PCR and Western blot, respectively. Bottom: Blot densities were quantified and presented as the means ± SEMs of 6–7 independent experiments. ^*^P < 0.05; ^**^P < 0.01 vs. corresponding value at 0 h. **(B)** Time-course of LTB4 production in vehicle- or HMGB1 (100 ng/ml)-stimulated BMDCs as determined by ELISA. Bottom: Color signals were quantified, and data were presented as the means ± SEMs of 6–7 independent experiments. ^*^P < 0.05; ^**^P < 0.01 vs. value at 0 h. **(C)** Representative immunocytochemical images of BMDCs stimulated with HMGB1. BMDCs were pre-treated with Zileuton at 10 or 30 μM for 1 h, and then stimulated with HMGB1 (100 ng/ml) for 10 days. Cells were stained with anti-CD11b/Mac-1 (red) and DAPI (blue), and then morphological changes and CD11b/Mac-1 expressions were photographed under a phase contrast microscope. Arrows indicate cells with a macrophage-like morphology. Right: Images were analyzed using Image J, and data were presented as the means ± SEMs of 3–4 independent experiments. ^**^P < 0.01 vs. control, ^##^*P* < 0.01 vs. vehicle.

The effects of HMGB1 on the expression of 5-LO mRNA and protein in BMDCs were determined using semi-quantitative RT-PCR and Western blot analysis. In previous studies, HMGB1 were secreted to 10–100 ng/ml physiologically or pathologically (27, 28). Thus, BMDCs were treated with HMGB1 at concentrations of 100 ng/ml in our study. As shown in Figure 1A, HMGB1 at concentration of 100 ng/ml increased the mRNA and protein expression of 5-LO in a time-dependent manner in BMDCs and THP-1 cells (Supplementary Figure 1), which were attenuated by inhibition of various receptors for HMGB1 (Supplementary Figure 2). To determine the functional role of 5-LO increased in HMGB1-stimulated cells, LTB4 production in HMGB1-treated cells was measured using ELISA. As shown in Figure 1B, LTB4 production in HMGB1-stimulated cells was gradually increased up to 24 h (approximately 10 ng/10^7^ cells), suggesting the potential involvement of 5-LO-derived LTs in MMD induction by HMGB1.

To evaluate the potential role for 5-LO on MMD induction by HMGB1, we determined the effects of Zileuton (10 or 30 μM), a 5-LO inhibitor, on MMD induced by HMGB1. When the cellular morphology of HMGB1-stimulated BMDCs were photographed under a phase contrast microscope, the majority of cells had a macrophage-like morphology, were larger than non-stimulated cells, and strongly adherent and irregular or spindle shaped. Immunocytochemistry of HMGB1-stimulated BMDCs also revealed that the surface expression of CD11b/Mac-1 (red) was markedly increased, which was significantly attenuated by pre-treatment with Zileuton, suggesting a potential role for 5-LO in MMD induced by HMGB1 (Figure 1C).

### Participation of BLTR1 signaling in monocytes during HMGB1-induced MMD

**Figure 2:**
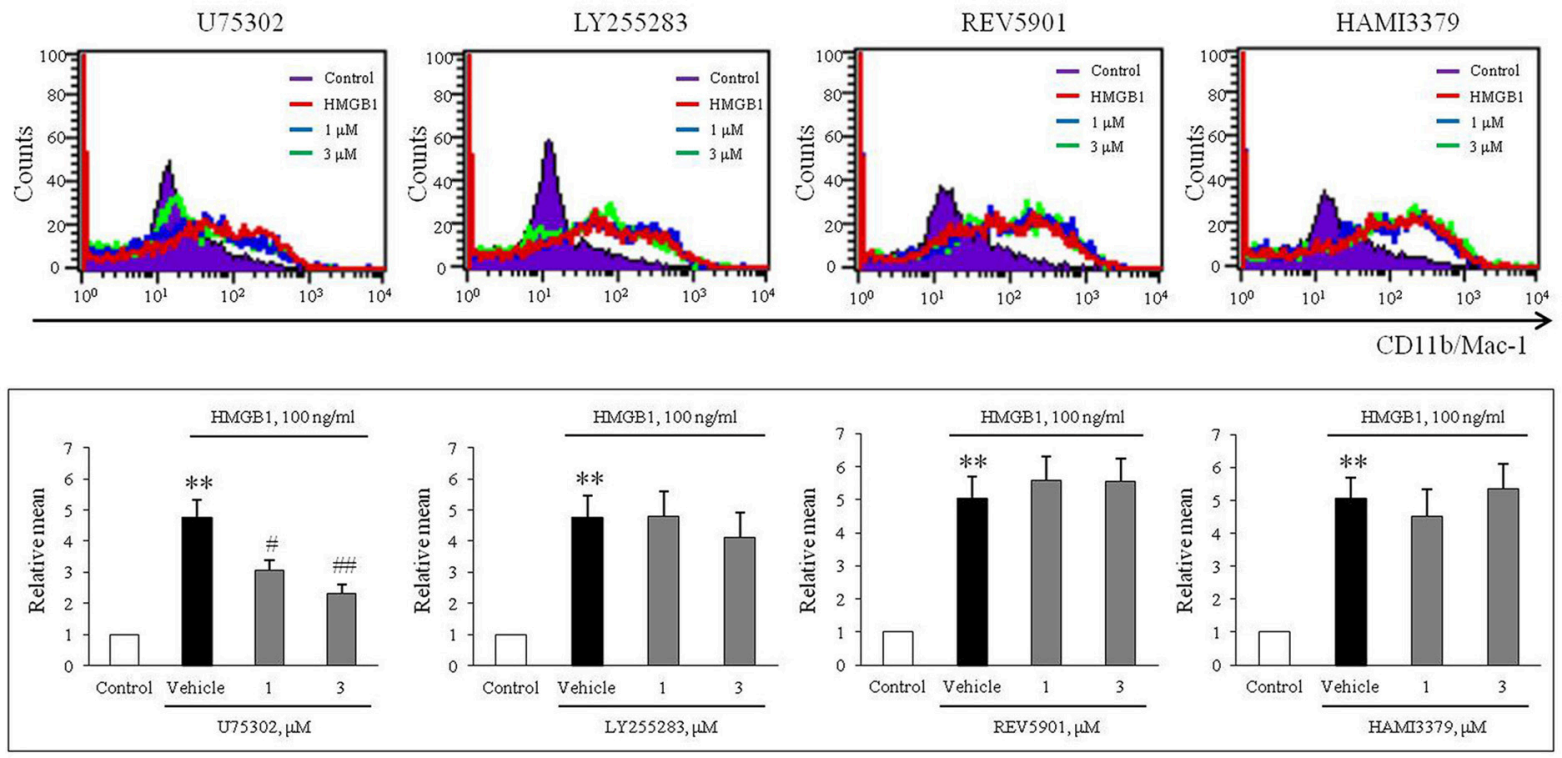
A potential role of BLTR1 signaling in monocytes on HMGB1-induced MMD. Representative flow cytometric images of CD11b/Mac-1 expression in BMDCs treated with HMGB1 (100 ng/ml) in the presence or absence of inhibitors for BLTR1 (U75302), BLTR2 (LY255283), cysLTR1 (REV5901), and cysLTR2 (HAMI3379). Bottom: Surface expressions of CD11b/Mac-1 on macrophages were expressed as mean fluorescent intensities. Quantified data were presented as the means ± SEMs of 7–8 independent experiments. ^**^P < 0.01 vs. control, ^#^*P* < 0.05; ^##^*P* < 0.01 vs. vehicle.

**Figure 3:**
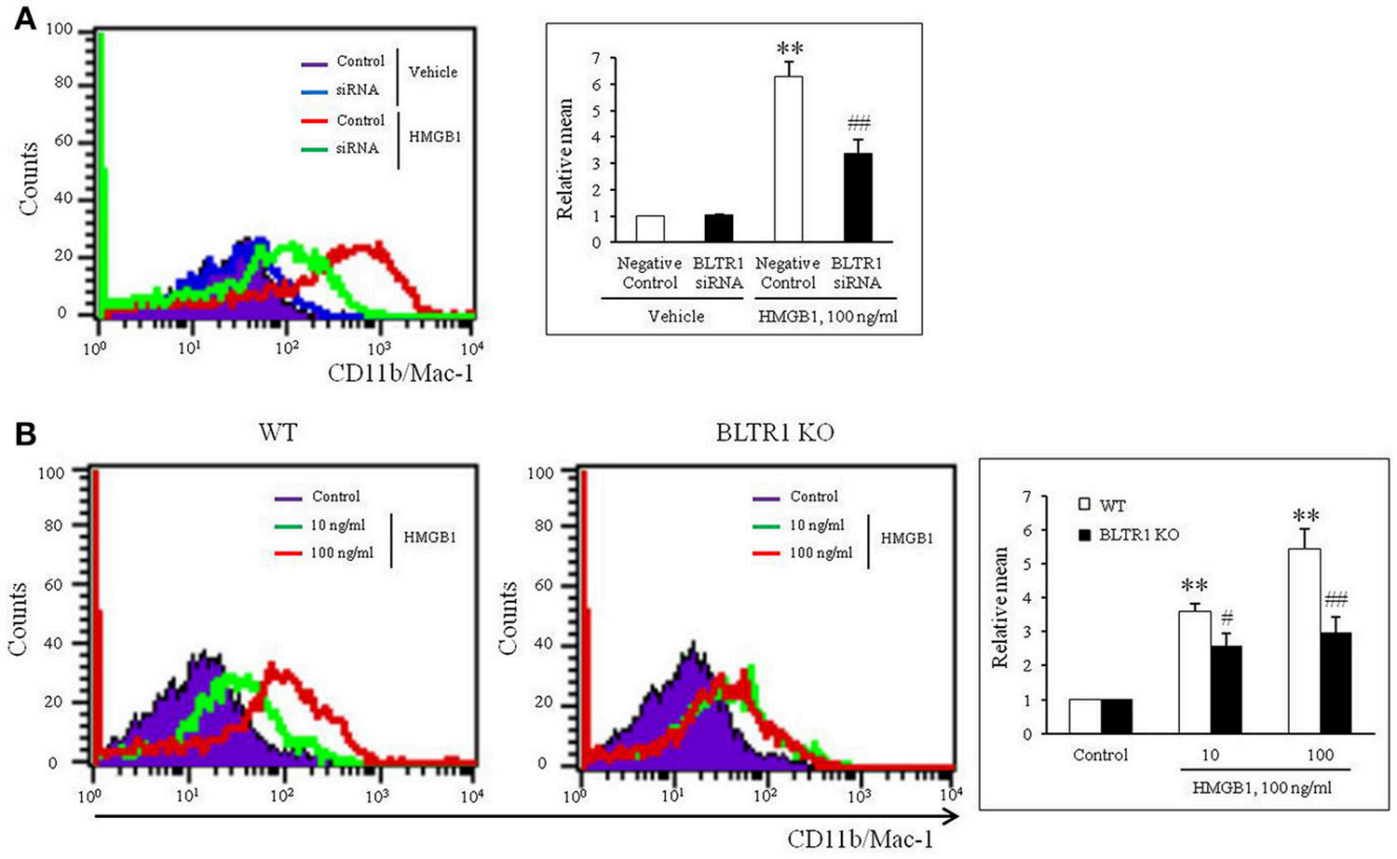
Identification of the role played by BLTR1 in monocytes during HMGB1-induced MMD. **(A)** Representative flow cytometric images of CD11b/Mac-1 expression in BLTR1-depleted BMDCs. BMDCs were transfected with negative control or BLTR1 siRNA for 48 h, and then stimulated with HMGB1 (100 ng/ml) for 10 days. Right: Mean fluorescent intensities were quantified, and data were presented as the means ± SEMs of 5–6 independent experiments. ^**^P < 0.01 vs. corresponding negative control in vehicle, ^##^*P* < 0.01 vs. negative control in HMGB1. **(B)** Representative flow cytometric images of CD11b/Mac-1 expression in BLTR1-deficient BMDCs. BMDCs isolated from wild-type (WT) or BLTR1-deficient (KO) mice were stimulated with HMGB1 for 10 days, and then cellular expressions of CD11b/Mac-1 were determined by flow cytometry. Right: Differential interface flow cytometric images of CD11b/Mac-1 expression were quantified, and data were presented as the means ± SEMs of 4–5 independent experiments. ^**^P < 0.01 vs. corresponding control, ^#^*P* < 0.05; ^##^*P* < 0.01 vs. corresponding value in WT mice.

To evaluate the role of leukotriene (LT) receptor signaling in monocytes during HMGB1-induced MMD, BMDCs were stimulated with HMGB1 (100 ng/ml) for 10 days in the presence of various leukotriene receptor inhibitors, including U75302 (a BLTR1 inhibitor), LY255283 (a BLTR2 inhibitor), REV5901 (a cysLTR1 inhibitor), and HAMI3379 (a cysLTR2 inhibitor). As shown in Figure 2, flow cytometric analysis showed an increase in the surface expression of CD11b/Mac-1 on BMDCs stimulated with HMGB1, which were attenuated dose-dependently by pretreatment with a BLTR1 inhibitor (U75302), but not by BLTR2 and cysLTR inhibitors. To further identify the role of BLTR1 in monocytes, we determined HMGB1-induced CD11b/Mac-1 expression in BLTR1-deficient BMDCs. As shown in Figure 3, HMGB1-induced expression of CD11b/Mac-1 on BMDCs was markedly attenuated in BLTR1-depleted cells using siRNA as well as in BLTR1-deficient cells isolated from BLTR1-deficient mice, suggesting a pivotal involvement of BLTR1 in HMGB1-induced MMD.

### Exogenous LTB4 augmented HMGB1-induced MMD in 5-LO-deficient BMDCs, but not in BLTR1-deficient BMDCs

**Figure 4:**
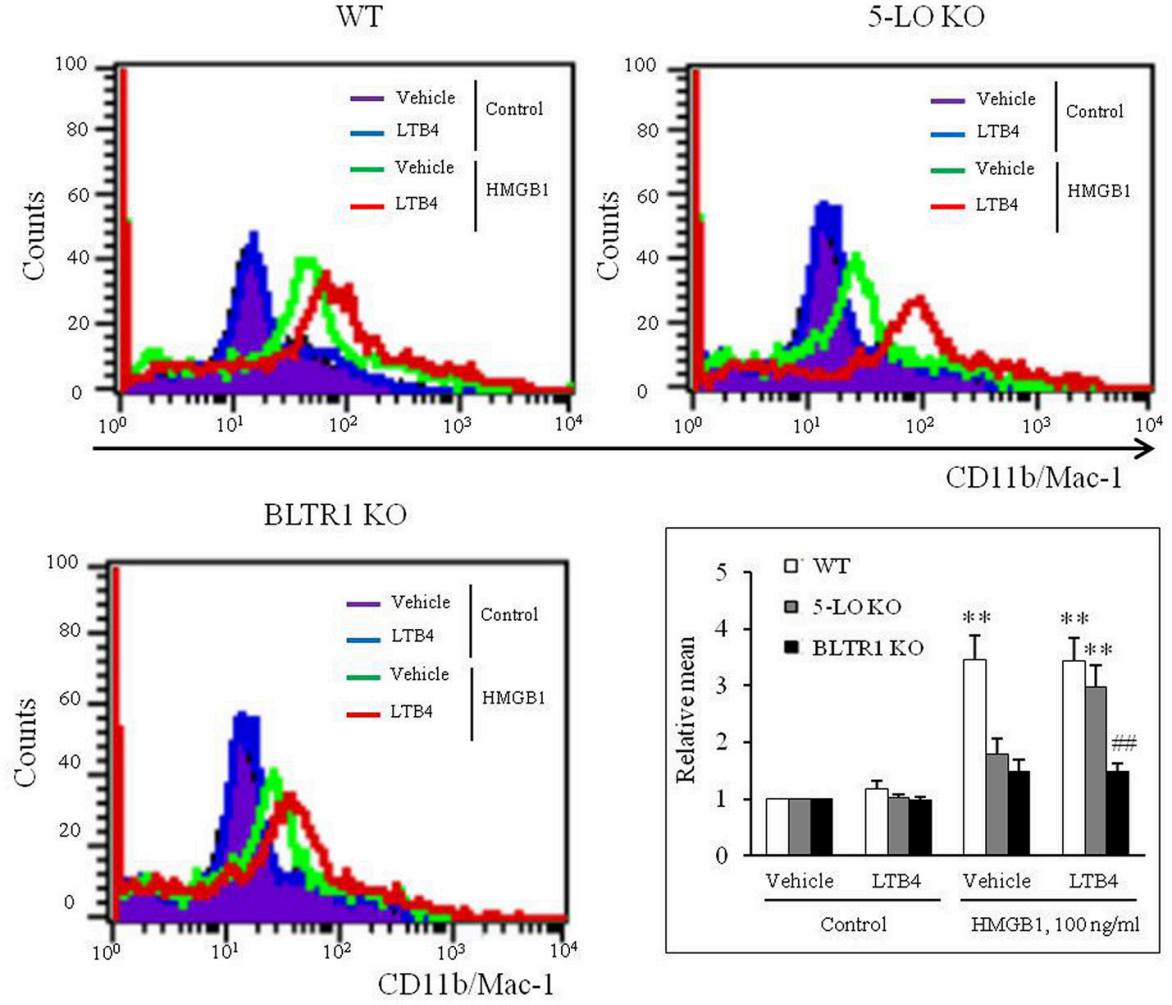
Role of LTB4-BLTR1 signaling in monocytes on HMGB1-induced MMD. Representative flow cytometric images of CD11b/Mac-1 expression in HMGB1-stimulated BMDCs in the presence or absence of exogenous LTB4. BMDCs isolated from wild-type (WT), 5-LO-deficient (KO), and BLTR1-KO mice, were incubated with LTB4 (10 ng/ml) for 1 h, and then treated with HMGB1 (100 ng/ml) to induce MMD. The cellular expressions of CD11b/Mac-1 were determined by flow cytometry. Bottom right: Differential CD11b/Mac-1 expressions in interface flow cytometric images were quantified, and data were presented as the means ± SEMs of 8–9 independent experiments. ^**^P < 0.01 vs. corresponding value in control, ^##^*P* < 0.01 vs. corresponding value in WT mice.

On the basis of the hypothesis that 5-LO-derived LTB4 in HMGB1-stimulated cells might play an important role in the process of MMD, the effects of exogenous LTB4 on MMD were investigated in 5-LO-deficient cells. Although LTB4 (1–10 ng/ml) had minimal direct effects on MMD in control and 5-LO-deficient BMDCs (Supplementary Figure 3), the attenuated MMD in 5-LO-deficient cells stimulated with HMGB1 was significantly reversed to the control level when cells were pre-treated with LTB4 at 10 ng/ml, a concentration comparable to that produced in HMGB1 (100 ng/ml)-stimulated control cells (Figure 4), which indicated the importance of the role played by LTB4 in HMGB1-induced MMD. Interestingly, the attenuated MMD by HMGB1 in BLTR1-deficient cells was not reversed by pre-treating cells with exogenous LTB4 (Figure 4). Collectively, these findings indicate the important role of LTB4-BLTR1 signaling in HMGB1-induced MMD in monocytes.

### Role of BLTR1 in vascular inflammation and neointima formation in wire-injured vasculature

**Figure 5:**
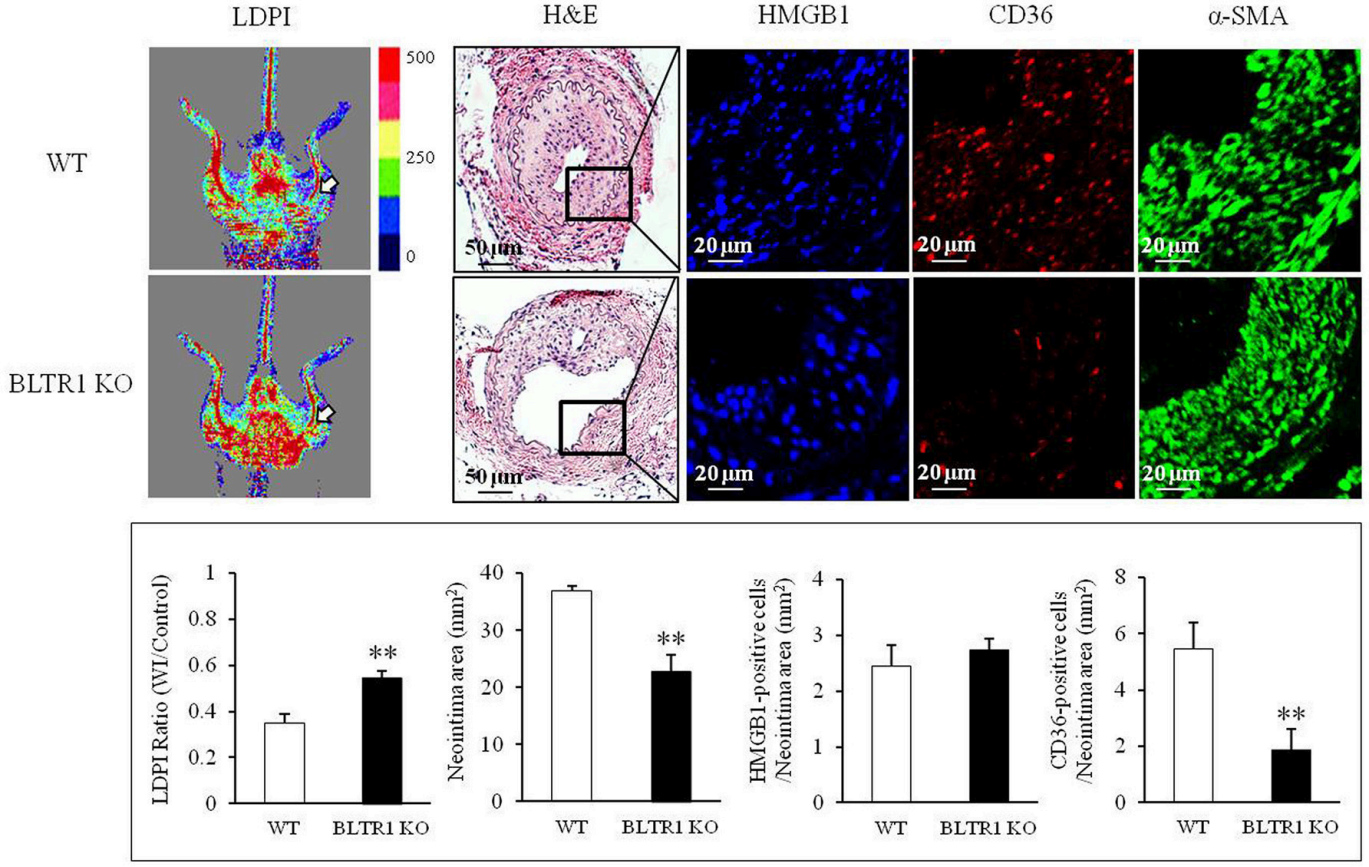
Involvement of BLTR1 in macrophage infiltration and neointima formation in wire-injured femoral arteries. Doppler images: Blood flow in the femoral arteries of WT and BLTR1-deficient (KO) mice at 4 wks after wire injury (WI) was assessed using a LDPI analyzer. In these color-coded images, red hue indicates regions of maximum perfusion, medium perfusion values are shown in yellow, and lowest perfusion values are represented as blue. Arrows indicate blood flow in an injured femoral artery. Photographs are representative of 5-6 independent experiments. H&E: Cross sections of mouse femoral arteries were prepared at 4 wks after WI, and stained with H&E. HMGB1 and CD36: HMGB1 and macrophage infiltration in the indicated neointima were stained with anti-HMGB1 antibody and anti-CD36 antibody, respectively. α-SMA: VSMCs were stained with anti-α-SMA antibody. Images are representative of 5–6 independent experiments. Bottom: LDPI ratio was quantified as the ratio of the blue-to-red pixels in the injured artery (WI) vs. non-injured arteries (Control). Neointima volumes in the cross sections of injured femoral artery were determined using an image analyzer. Numbers of HMGB1-positive and CD36-positive cells in neointima area were quantified, and data were presented as the means ± SEMs of 3-4 independent experiments. ^**^P < 0.01 vs. WT mice.

To investigate the potential involvement of HMGB1 in the progression of vascular inflammation and neointima formation, we determined the levels of HMGB1 in the injured vasculatures. At 4 wks after wire injury, HMGB1 levels were markedly increased in neointima lesions of the injured vasculatures from both control and BLTR1-deficient mice. However, blood flow changes and neointima formation in the injured vasculatures were attenuated in BLTR1-deficient mice compared to those of control mice. Likewise, macrophage infiltration into neointima was also markedly attenuated in BLTR1-deficient mice (Figure 5), suggesting BLTR1 contributed to vascular inflammation and subsequent neointima formation induced by damage-associated mediators secreted in the injured vasculatures.

**Figure 6:**
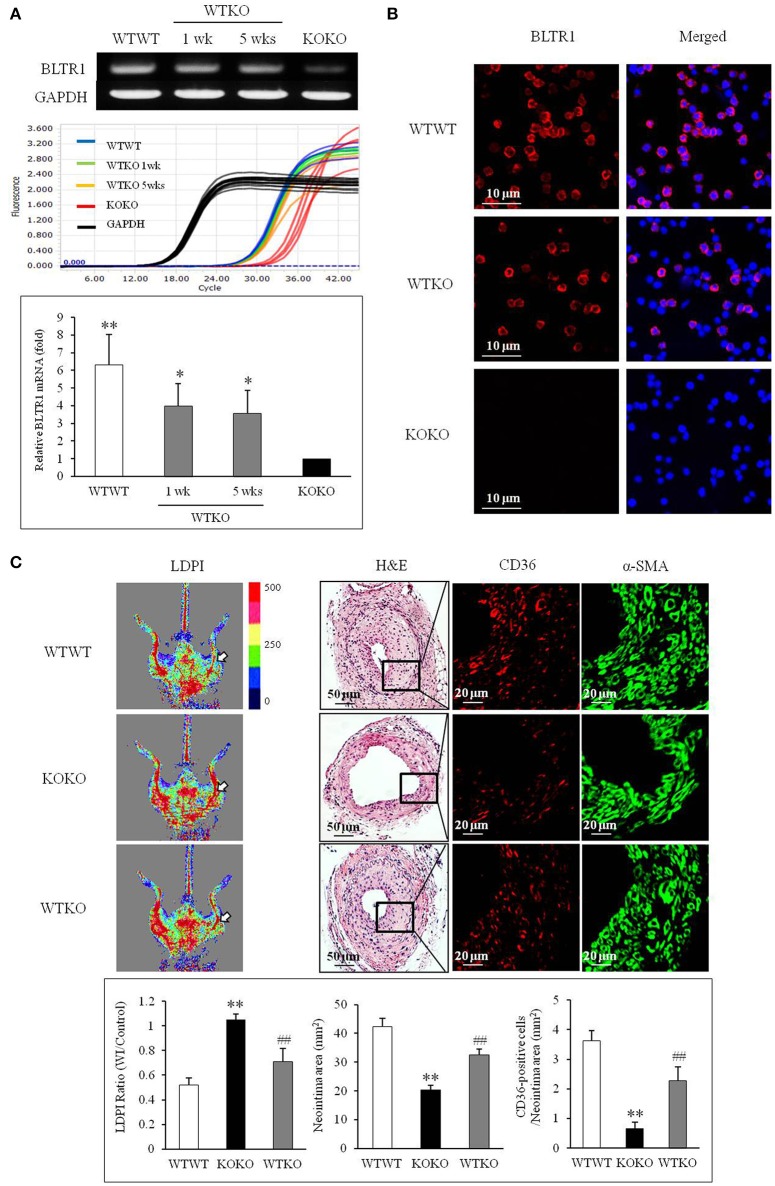
Identification of a pivotal role for BLTR1 in monocytes on vascular inflammation and neointima formation in the wire-injured femoral artery. **(A)** Expressions of BLTR1 mRNA in PBMCs isolated from three groups of mice transplanted with BMDMs (WTWT, WT cells into WT mice; KOKO, BLTR1-deficient cells into BLTR1-deficient mice; and WTKO, WT cells into BLTR1-deficient mice) were determined by Real Time PCR. PCR signals were quantified, and data were presented as the means ± SEMs of 4–5 independent experiments. ^*^P < 0.05; ^**^P < 0.01 vs. KOKO mice. **(B)** Expressions of BLTR1 protein in PBMCs isolated from these three groups of mice at 5 wks post-transplantation were determined by immunocytochemistry. Photographs are representative of 3–4 independent experiments. **(C)** Doppler imaging: Blood flow in the femoral arteries of WTWT, WTKO, and KOKO mice at 4 wks after wire injury were monitored using a LDPI analyzer. Arrows indicate blood flow in the injured femoral artery. Photographs are representative of 4–5 independent experiments. H&E: Cross sections of the femoral arteries of WTWT, WTKO, and KOKO mice at 4 wks after wire injury were stained with H&E. CD36 & α-SMA: Infiltrating macrophages in indicated neointima was stained with anti-CD36 antibody, and VSMCs were stained with anti-α-SMA antibody. Photographs are representative of 4–5 independent experiments. Bottom: LDPI ratio was quantified as the ratio of the blue-to-red pixels in the injured artery (WI) vs. non-injured arteries (Control). Neointima volumes were determined using an image analyzer. Numbers of CD36-positive cells within neointima area in the injured artery of WT and BLTR1-deficient mice were quantified, and data were expressed as the means ± SEMs of 4–5 independent experiments. ^**^P < 0.01 vs. WTWT mice, ^##^*P* < 0.01 vs. KOKO mice.

To investigate the contribution of BLTR1 in monocytes to macrophage infiltration of neointima lesions, BMDMs of WT mice were adoptively transferred into BLTR1-deficient mice. When BLTR1 mRNA levels in PBMCs isolated from the three groups of BMDM-transplanted mice (WTWT, WT monocytes into WT mice; WTKO, WT monocytes into BLTR1-deficient mice; and KOKO, BLTR1-deficient monocytes into BLTR1-deficient mice) were determined by Real Time PCR, an increase in BLTR1 mRNA levels in the monocytes of recipient mice was detected at 1 and 5 wks after adoptive transplantation (Figure 6A). Likewise, an increase in BLTR1 protein levels observed in monocytes of recipient mice was also detected at 5 wks after adoptive transplantation (Figure 6B). As shown in Figure 6C, intimal hyperplasia and macrophage infiltration were significantly increased in WT monocyte-recipient mice (WTKO) comparing with that in BLTR1-deficient mice transferred with BLTR1-deficient BMDMs (KOKO). These observations suggested that BLTR1 in monocytes played a critical role in the infiltration of macrophage into neointima lesions, and that they influenced neointima formation in our murine model of femoral artery injury.

## Discussion

In this study, we investigated the importance of leukotriene signaling in monocytes on monocyte-to-macrophage differentiation and vascular inflammation and resultant intimal hyperplasia in a mouse model of wire-injured femoral artery. In cultured primary BMDCs, genetic or pharmacological inhibition of the 5-lipoxygenase pathway in monocytes attenuated MMD induced by HMGB1, an endogenous damage-associated molecular patterns. Among various inhibitors for leukotriene receptors, U75302, a BLTR1 inhibitor, exclusively attenuated MMD induced by HMGB1. The importance of BLTR1 signaling during HMGB1-induced MMD was also demonstrated using BLTR1-deficient BMDCs. Thus, BLTR1 signaling in monocytes was suggested as a pivotal player in MMD induced by HMGB1, leading to vascular inflammation after vascular injury.

MMD is a key event in the process of vascular inflammation, which results in the remodeling of the injured vasculatures (29). Thus, an understanding of the fundamental molecular mechanisms that underlie this differentiation is an important aspect of identifying new therapeutic strategies. In our previous study, the importance of 5-LO in monocytes was identified using genetic and pharmacological inhibition of the 5-LO pathway in monocytes (7). In addition, in accordance with previous report by Yu et al. (18) in which disruption of the LT synthesis/response pathway in myeloid cells restrained several components of response to injury, we showed in a previous *in vivo* study 5-LO in monocytes played a pivotal role in vascular inflammation and resultant restenosis (7). However, although the importance of 5-LO in monocytes during MMD with subsequent vascular inflammation was identified, the 5-LO-linked signaling in monocytes mediating MMD need to be identified to develop specific target-based therapeutics, because 5-LO in monocytes exert its action via production of various leukotrienes (LT) including LTB4 and cysteinyl LTs.

Leukotrienes exert their actions via four subclasses of 7-transmembrane G-protein-coupled cell surface receptors. BLTR1 and BLTR2 are high and low affinity receptors of LTB4, respectively, whereas CysLT1 and CysLT2 are activated by cysteinyl-LTs (30–32). Thus, we stimulated BMDCs in the presence of various inhibitors for LT receptors including LTB4 receptors (BLTR1 or BLTR2) or cysteinyl LT receptors (cysLTR1 or cysLTR2). In our present study, U75302 (a BLTR1 inhibitor) exclusively attenuated MMD induced by HMGB1 among various inhibitors. The importance of BLTR1 in monocytes on HMGB1-induced MMD was also demonstrated using BLTR1-deficient cells. Moreover, HMGB1 increased 5-LO expression in monocytes with subsequent production of LTB4 in the present study, suggesting that HMGB1 might induce MMD via production of 5-LO-mediated production of LTB4.

To determine the direct functional role of exogenous LTB4 on MMD, BMDCs were stimulated with various concentrations of LTB4 in the absence or presence of HMGB1. We found that LTB4 had minimal effects on MMD in cells from control, 5-LO-deficient and BLTR1-deficient mice. However, MMD suppression by HMGB1 was significantly reversed by exogenous LTB4 in 5-LO-deficient cells, but not in BLTR1-deficient cells, suggesting LTB4/BLTR1 signaling as a pivotal player for HMGB1-induced MMD. Thus, LTB4/BLTR1-mediated priming of monocytes is considered as an essential prerequisite for HMGB1-induced MMD based on the previous report that exogenous LTB4 potentiated the priming effect of cytokines on human monocytes (33). However, further studies are remained to determine the precise roles of these signals in cell priming.

The molecular processes that initiate inflammation in arterial walls after mechanical injury are not fully understood. Recently, endogenous molecules released during cell death and stress, termed DAMPs, could activate pattern recognition receptors and lead to inflammation, because endoluminal vascular interventional procedures cause stretching of vessel walls and subsequent cell necrosis (34). Thus, coordinated relationships exist between local vascular injury and pattern recognition receptor-related signals in the process of vascular inflammation (20). Of the various DAMPs, HMGB1 has emerged as an important regulator of inflammatory responses resulting from tissue injury (3, 4), and has been implicated as an active player in vascular inflammation leading to intimal hyperplasia after arterial injury (6). Reportedly, HMGB1 is known to have pro-inflammatory cytokine-like activity, promote chemotaxis, and stimulate cellular migration and growth (35). Interestingly, in a previous study we found HMGB1 enhanced MMD and caused vascular inflammation (7). Collectively, these observations suggest that HMGB1-related signals in monocytes might be considered therapeutic targets for the treatment of vascular inflammation.

In our *in vivo* study, both macrophage infiltration and intimal hyperplasia in the wire-injured femoral artery were markedly attenuated in BLTR1-deficient mice compared to that in wild-type control mice. Although the importance of BLTR1 in vascular smooth muscle cells in the intimal hyperplasia has been reported previously (16), we expected the potential role for BLTR1 in monocytes in vascular inflammation on the basis of our *in vitro* data in which the BLTR1 signaling in monocytes played a pivotal role in MMD induced by HMGB1. To confirm the contribution of BLTR1 in monocytes in macrophage infiltration into neointimal lesion, BMDMs isolated from WT mice were adoptively transferred into BLTR1-deficient mice as previously described (7, 18). In our present study, intimal hyperplasia and macrophage infiltration were significantly greater in BLTR1-deficient mice administered WT BMDMs than those in BLTR1-deficient mice administered BLTR1-deficient BMDMs. Based on these results, it was suggested that BLTR1 in monocytes played a pivotal role in MMD induced by HMGB1, and subsequent macrophage infiltration in the injured vasculatures with neointima formation in our murine wire-injured femoral artery model.

## Author contributions

SEB and CDK designed and performed experiments, analyzed the experimental data, and wrote the manuscript, and SSB, KK, and WSL contributed to data analysis. SYP performed experiments. All authors approved the final manuscript.

### Conflict of interest statement

The authors declare that the research was conducted in the absence of any commercial or financial relationships that could be construed as a potential conflict of interest.

## Abbreviations

HMGB1: High mobility group box 1 protein
5-LO: 5-Lipoxygenase
MMD: monocyte to macrophage differentiation
BMDC: Bone marrow derived cell
BLTR: receptor for leukotriene B4
CysLTR: receptor for cysteinyl-leukotrienes
DAMP: Damage-associated molecular patterns
VSMC: Vascular smooth muscle cell
FLAP: 5-Lipoxygenase activating protein
LT: Leukotriene
LTB4: Leukotriene B4
BMDM: Bone marrow derived monocyte
PBMC: Peripheral blood mononuclear cell
LDPI: Laser Doppler perfusion imaging
H&E: Hematoxylin and Eosin
α-SMA: alpha-smooth muscle actin
DAPI: 4′6-Diamidino-2-phenylindole
WT: Wild-type.
