# Supplementary material for: BLTR1 in Monocytes Emerges as a Therapeutic Target For Vascular Inflammation With a Subsequent Intimal Hyperplasia in a Murine Wire-Injured Femoral Artery
Source: Front Immunol. 2018 Aug 28;9:1938. doi: 10.3389/fimmu.2018.01938 (PMC6121004; doi:10.3389/fimmu.2018.01938)
Supplement: Supplementary file 1 [file Data_Sheet_1.docx]

**Supplementary Material**

**Materials and Methods**

**Cell Culture**

THP-1 cells (a human monocytic leukemia cell line) were purchased from the ATCC (Manassas, VA, USA). Cells were grown in RPMI 1640 medium (Life Technologies, Carlsbad, CA, USA) supplemented with 10% heat-inactivated fetal bovine serum (FBS), antibiotic-antimycotic, and L-glutamine (Life Technologies, Carlsbad, CA, USA), and maintained at 37 ℃ in a humidified 5% CO_2_/95% air atmosphere.

**Chemicals and Antibodies**

5-LO, β-actin, TLR2, TLR4 and RAGE antibodies were purchased from Santa Cruz Biotechnology (Santa Cruz, CA, USA), R-phycoerythrin PE-conjugated mouse anti-human CD11b/Mac-1 antibody and PE-conjugated mouse IgG isotype control antibody were from BD (San Diego, CA, USA). Horseradish peroxidase (HRP)-conjugated IgG antibody (Santa Cruz Biotechnology) was used as the secondary antibody. PCR primers were from Bioneer (Daejeon, ROK). LTB4 was from Cayman Chemical Inc (Ann Arbor, MI, USA).

**Western Blot Analysis**

THP-1 cell lysates were prepared in ice-cold lysis buffer, and equal amounts of proteins were separated on 10% polyacrylamide gel under reducing conditions, and then transferred to nitrocellulose membranes (Amersham-Pharmacia, Piscataway, NJ, USA). Membranes were blocked with 5% skim milk in TBST and incubated overnight with primary antibody (1:1000) in 5% skim milk. Blots were washed with TBST, incubated with HRP-conjugated secondary antibody for 2 hrs, and developed using ECL Western blot detection reagents (Amersham-Pharmacia). Membranes were re-blotted with anti-β-actin antibody (Santa Cruz Biotechnology) as an internal control. Signals from bands were quantified using US-SCAN-IT gel 5.1 software (Silk Scientific, Orem, Utah, USA). Data were expressed as relative densities.

**Reverse Transcription-PCR (Polymerase Chain Reaction) Analysis**

Total RNA was isolated from cells using QIAzol (Qiagen, Hilden, Germany) and reverse transcribed into cDNA using the Improm-II Reverse Transcription System (Promega, Madison, WI, USA). cDNA amplification was performed using primers specific for 5-LO (forward, 5'-AGTACCTGACCGTGGTGATCTTCA-3'; reverse, 5'-TCAGATGGCCACACTGTTCGGAAT-3'). 5-LO mRNA levels in THP-1 cells were quantified by RT-PCR using GAPDH mRNA as the internal standard. Relative intensities were expressed as fold changes versus GAPDH.

**Small Interfering RNA (siRNA) Preparation and Transfection**

TLR2, TLR4 and RAGE siRNA oligonucleotides were synthesized by Bioneer. The siRNA negative control duplex was used as a control oligonucleotide. siRNA molecules were transfected into cells using Lipofectamine 2000 (Invitrogen, Carlsbad, CA, USA) according to the manufacturer’s instructions.

**Statistical Analysis**

Results were expressed as means ± SEMs. One-way analysis of variance (ANOVA) followed by Turkey’s multiple comparison test was used to determine the significance of differences. Statistical significance was accepted for *P* values < 0.05.

**Supplementary Figures**

**Supplementary Figure 1. Characteristics of 5-LO expression in THP-1 cells stimulated with HMGB1.**

THP-1 cells (a human monocytic leukemia cell line) were stimulated with 100 ng/ml of HMGB1 for the indicated times, and 5-LO mRNA and protein levels were then assessed by RT-PCR and Western blotting, respectively. Relative intensities of 5-LO mRNA and protein versus GAPDH and β-actin were quantified, and data were presented as the means ± SEMs of 4-5 independent experiments. **P* < 0.05; ***P* < 0.01 vs. value at 0 hr.

**Supplementary Figure 2. Involvement of TLR2, TLR4 and RAGE in HMGB1-induced 5-LO expression in THP-1 monocytes.**

**(A)** Representative Western blots of 5-LO expression in THP-1 cells stimulated with HMGB1. THP-1 cells were pretreated with anti-IgG antibody (10 µg/ml), anti-TLR2 antibody (10 µg/ml), anti-TLR4 antibody (10 µg/ml), and anti-RAGE antibody (10 µg/ml) for 30 min, and then stimulated with HMGB1 (100 ng/ml) for 24 hr. Bottom: Blot densities were quantified, and data were presented as the means ± SEMs of 4-5 independent experiments. ***P* < 0.01 vs. Control, ^##^*P* < 0.01 vs. Vehicle. **(B)** THP-1 cells were transfected with siRNAs (200 nM) for TLR2, TLR4 and RAGE, and then stimulated with HMGB1 (100 ng/ml) for 24 hr. Right: Relative intensities of 5-LO expression were quantified, and data were expressed as the means ± SEMs of 4-5 independent experiments. ***P* < 0.01 vs. corresponding control, ^##^*P* < 0.01 vs. negative control.

**Supplementary Figure 3. Effects of exogenous LTB4 on MMD.**

Representative flow cytometric images of CD11b/Mac-1 expression in BMDCs isolated from WT, 5-LO-deficient (KO) and BLTR1-KO mice. Cells were stimulated with LTB4 (1-10 ng/ml) for 10 days. Mean fluorescent intensities were quantified, and data were presented as the means ± SEMs of 5-6 independent experiments.
