# Supplementary figures and images for: BLTR1 in Monocytes Emerges as a Therapeutic Target For Vascular Inflammation With a Subsequent Intimal Hyperplasia in a Murine Wire-Injured Femoral Artery
Source: Front Immunol. 2018 Aug 28;9:1938. doi: 10.3389/fimmu.2018.01938 (PMC6121004; doi:10.3389/fimmu.2018.01938)

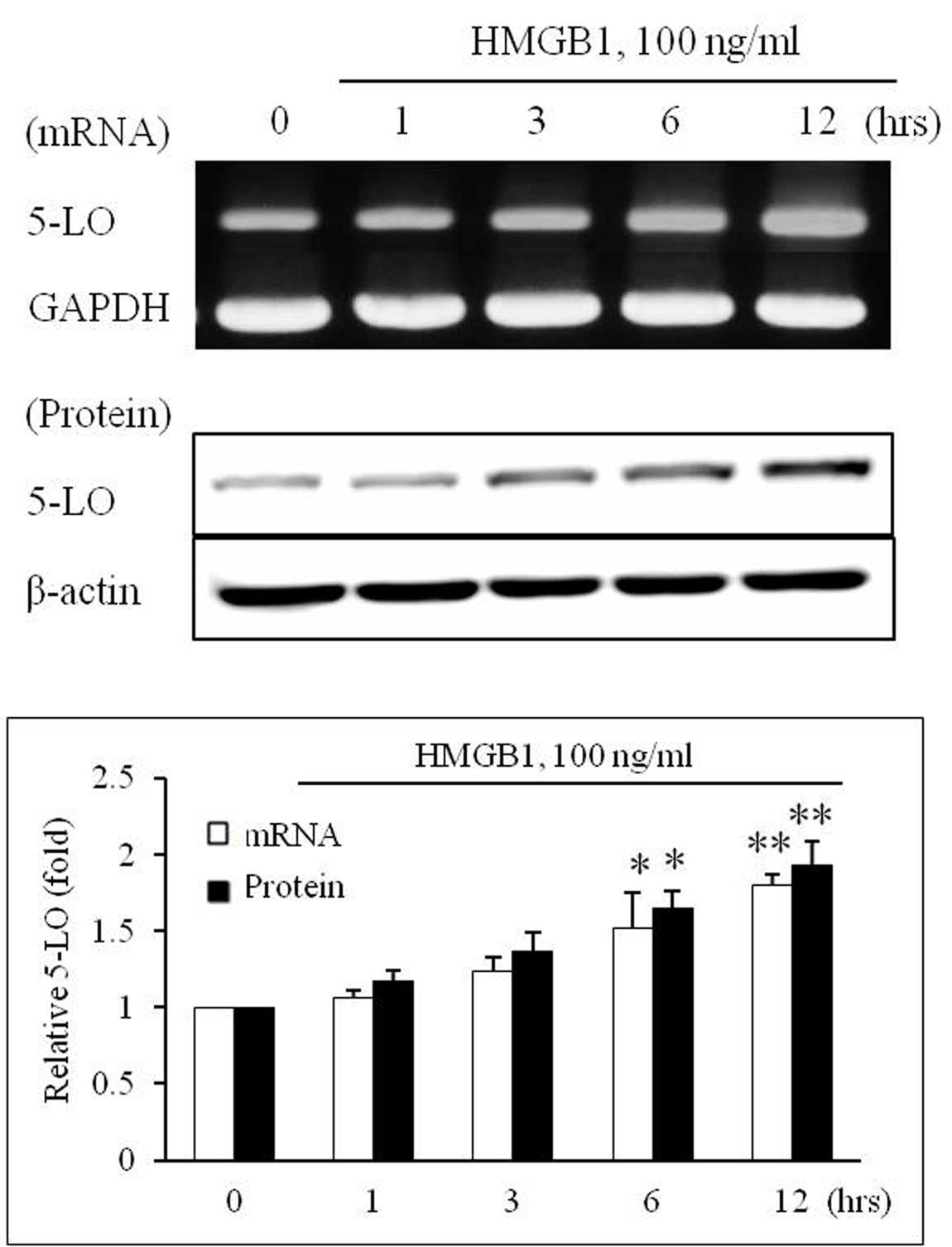

Supplement: Supplementary file 2 [file Image_1.TIF]

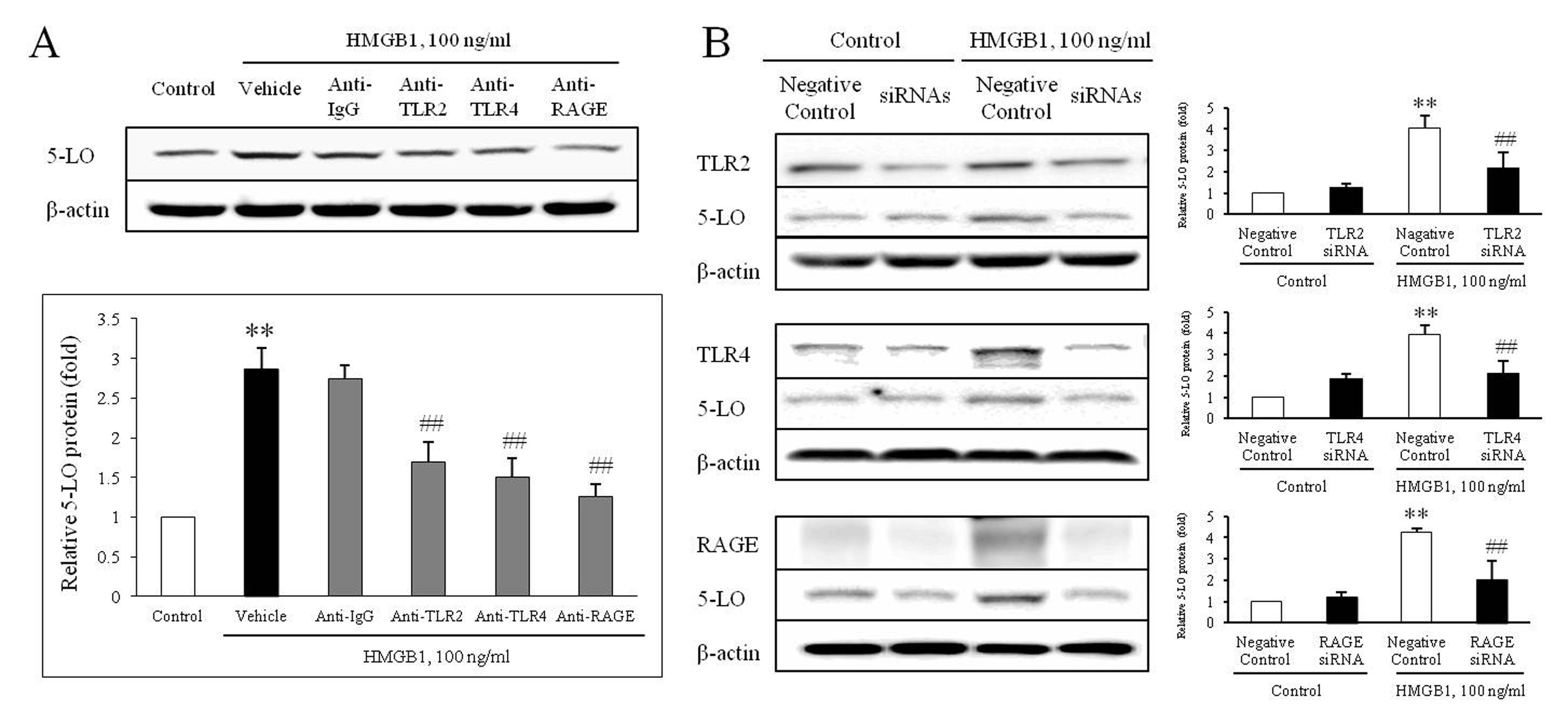

Supplement: Supplementary file 3 [file Image_2.TIF]

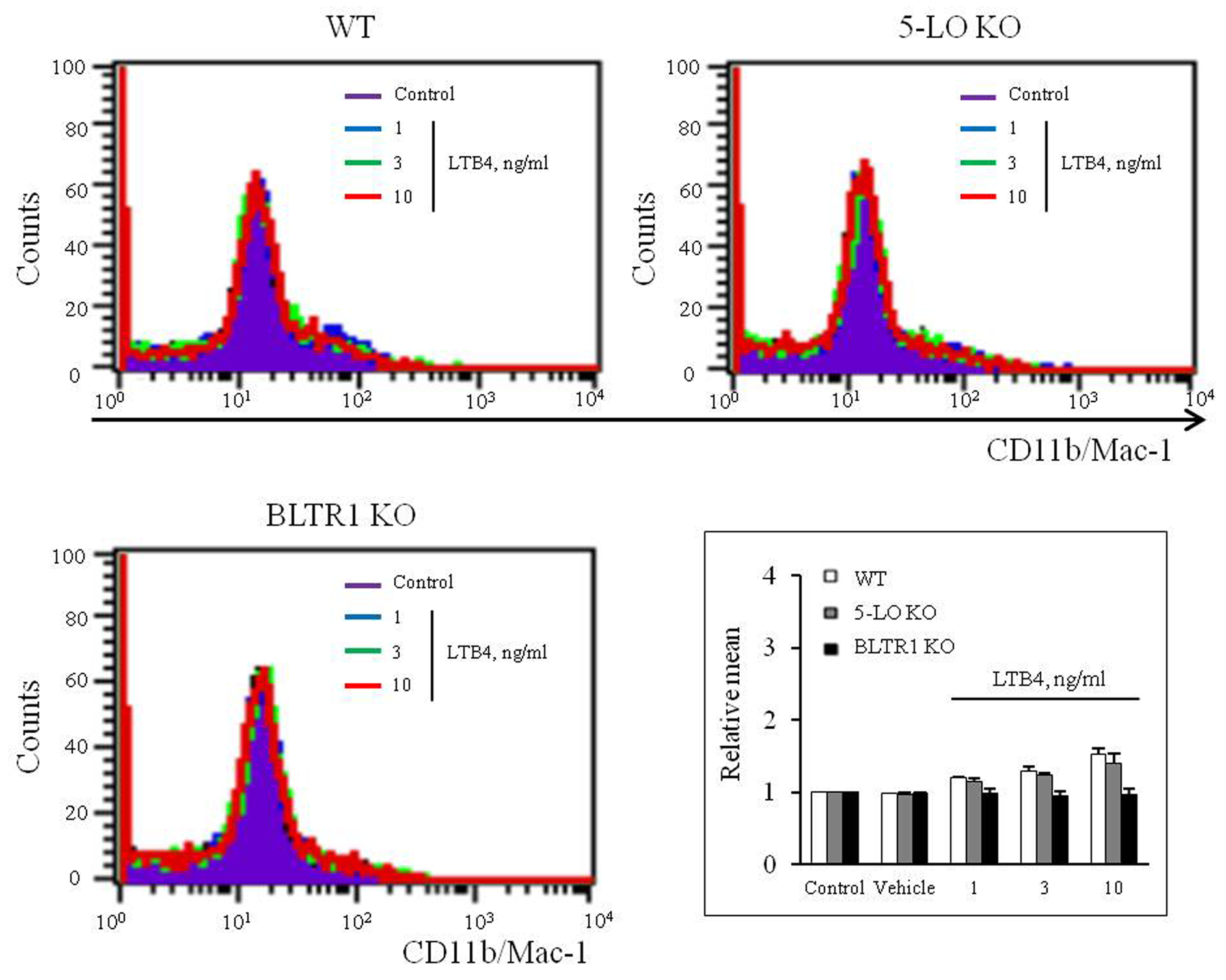

Supplement: Supplementary file 4 [file Image_3.TIF]
